# Rural and urban differences in health system performance among older Chinese adults: cross-sectional analysis of a national sample

**DOI:** 10.1186/s12913-020-05194-6

**Published:** 2020-05-04

**Authors:** Vicky Mengqi Qin, Barbara McPake, Magdalena Z. Raban, Thomas E. Cowling, Riyadh Alshamsan, Kee Seng Chia, Peter C. Smith, Rifat Atun, John Tayu Lee

**Affiliations:** 1 Saw Swee Hock School of Public Health, National University of Singapore, Singapore, Singapore; 2 Nossal Institute for Global Health, University of Melbourne, Melbourne, Victoria Australia; 3 Centre for Health Systems and Safety Research, Australian Institute of Health Innovation, Macquarie University, Sydney, New South Wales Australia; 4 Department of Health Services Research and Policy, Faculty of Public Health and Policy, London School of Hygiene & Tropical Medicine, London, UK; 5 Centre for Health Economics, University of York, York, UK; 6 Imperial College London, London, UK; 7 Department of Global Health and Population, Harvard T.H. Chan School of Public Health, Harvard University, Cambridge, MA USA; 8 Department of Global Health and Social Medicine, Harvard Medical School, Harvard University, Boston, MA USA; 9 Department of Primary Care and Public Health, School of Public Health, Imperial College, London, UK

**Keywords:** Health system performance, Health systems, Rural-urban disparity, Health policy, China

## Abstract

**Background:**

Despite improvement in health outcomes over the past few decades, China still experiences striking rural-urban health inequalities. There is limited research on the rural-urban differences in health system performance in China.

**Method:**

We conducted a cross-sectional analysis to compare health system performance between rural and urban areas in five key domains of the health system: effectiveness, cost, access, patient-centredness and equity, using data from the WHO Study on Global AGEing and adult health (SAGE), China. Multiple logistic and linear regression models were used to assess the first four domains, adjusting for individual characteristics, and a relative index of inequality (RII) was used to measure the equity domain.

**Findings:**

Compared to urban areas, rural areas had poorer performance in the management and control of hypertension and diabetes, with more than 50% lower odds of having breast (AOR = 0.44; 95% CI: 0.30, 0.64) and cervical cancer screening (AOR = 0.49; 95% CI: 0.29, 0.83). There was better performance in rural areas in the patient-centredness domain, with more than twice higher odds of getting prompt attention, respect, clarity of the communication with health provider and involvement in decision making of the treatment in inpatient care (AOR = 2.56, 2.15, 2.28, 2.28). Although rural residents incurred relatively less out-of-pocket expenditures (OOPE) for outpatient and inpatient services than urban residents, they were more likely to incur catastrophic expenditures on health (AOR = 1.30; 95% CI 1.16, 1.44). Wealth inequality was found in many indicators related to the effectiveness, costs and access domains in both rural and urban areas. Rural areas had greater inequalities in the management of hypertension and coverage of cervical cancer (RII = 7.45 vs 1.64).

**Conclusion:**

Our findings suggest that urban areas have achieved better prevention and management of non-communicable disease than rural areas, but access to healthcare was equivalent. A better understanding of the causes of the observed variations is needed to develop appropriate policy interventions which address these disparities.

## Background

Since the economic liberalization which began in the 1980s, China has experienced rapid economic growth (more than a 30-fold increase in gross national income per capita from US$220 in 1980 to US$8690 in 2017, at current US$) and improvements in health outcomes (average life expectancy at birth increased by more than 8 years in 2015) [1, 2]. However, these national averages obscure gross rural-urban inequities in outcomes [3, 4]. Compared to urban areas, under-5 mortality was more than twice higher (12.9 vs 5.8 per 1000 livebirths) in rural areas in 2015 [1, 5] The observed inequities between rural and urban areas in health outcomes may be linked to the different performance of the health system [6–11].

The government of China, via its Healthy China 2030 Plan and through a series of far-reaching health reforms, [12, 13] has set a bold vision for achieving universal health coverage (UHC) and reducing health inequalities. While almost the entire Chinese population (around 1.2 billion, over 95%) is currently covered by one of the three major social health insurance schemes (the urban employee basic medical insurance (UEBMI) scheme, the urban resident basic medical insurance (URBMI) scheme, and the new cooperative medical scheme (NCMS)), [4, 14] low levels of service coverage and high out-of-pocket expenditures have raised concerns about lack of financial protection for patients with major long-term health conditions. To ensure equitable access to health services, since 2014, social health insurance reforms have been focusing on transforming from dual to integrated health insurance system [15]. This includes long-term efforts at vertical consolidation (rural NCMS and urban URBMI and UEBMI) and horizontal consolidation (fund pools and benefit packages) [15, 16].

Understanding rural-urban differences in health system performance in China received increasing focus along with the policy initiative of UHC [17]. In China, inequalities between rural and urban areas may be exacerbated due to the residency control that further restricts population mobilization and deters cross-region access health care [10, 18]. Given the differences of China’s health system across regions, understanding variation in health system performance between rural and urban areas in China is highly relevant not only to identify areas of relatively good and poor performance, but also for improving resource allocation and for the development of effective and targeted policy interventions [19, 20]. This study aims to assess rural-urban differences in health system performance across a list of key indicators for elderly in China.

## Methods

### Sample and data

We used cross-sectional data from the World Health Organization (WHO) Study on Global Ageing and Adult Health (SAGE, wave 1). SAGE China adopted a stratified multistage cluster sampling strategy to generate the sample. The survey primarily includes a nationally representative cohort of persons aged 50 years and above, with a smaller cohort of persons aged 18 to 49 for comparison purposes [21]. In brief, the aim of the SAGE is to understand disability, healthcare utilisation and subjective well-being of adult populations to inform evidence based policy making. Rural/urban division was defined throughout the sampling process by SAGE. More details on the SAGE China sampling methods and assessment have been described elsewhere [22].

The sample size consists of a total of 15,050 adults aged 18 years and above from 10,278 households [22]. Our analysis was conducted based on the sample of respondents aged 50 years and older, which included the majority of the study population. The only exceptions to this were the analyses of breast and cervical cancer screening coverage, which comprised women in the appropriate age ranges for cancer screening, i.e. 50–74 years old for breast screening, and aged 25–69 years for cervical screening programs [23].

### Analytical framework and health system performance indicators

Our analyses adopted the health system framework from Arah el al [24]. which considers five key performance domains, including (a) effectiveness, (b) access, (c) patient-centredness, (d) cost, and (e) equity. This framework has been commonly used in other studies that evaluate comparative health system performance across countries [19, 24, 25]. A full list of the indicators and definitions are presented in Additional file 1.

#### Effectiveness

In the effectiveness domain, we constructed eleven indicators relating to the prevention and management of common chronic conditions, in the areas of: (1) management of hypertension; (2) management of diabetes; (3) medical treatment for depression; (4) breast cancer screening; (5) cervical cancer screening; (6) eye care; and (7) dental care. Respondents self-reported the management and prevention services of common chronic conditions they received. In the case of hypertension, respondents were considered to have hypertension if they self-reported having a diagnosis of hypertension, or if they had a mean systolic blood pressure of ≥140 mmHg or diastolic blood pressure of ≥90 mmHg. Those respondents identified as having hypertension, but not self-reporting the condition, were defined as being undiagnosed hypertensive. Controlled hypertension was defined as those who had hypertension but having a mean systolic blood pressure of ≤140 mmHg and diastolic blood pressure of ≤90 mmHg.

The assessment of coverage of cancer screening was based on the responses to the following questions from the SAGE: “When was the last time you had a mammography?” and “When was the last time you had a pelvic examination?” Respondents who answered positively for pelvic examination were then asked: “The last time you had a pelvic examination, did you have a Pap smear test?” Following commonly used guidelines for breast and cervical cancer screening in LMICs, coverage of breast cancer screening was defined as the percentage of women aged 50–74 years old who had a mammography in the past 3 years, and coverage of cervical cancer screening was defined as the percentage of women aged 25–69 years old who had a pap smear in the past 3 years [23].

#### Cost

Indicators included in the cost domain for outpatient and inpatient care were: 1) the percentage of respondents who reported that their last visit was free; 2) the mean and median out-of-pocket expenditure (OOPE) amounts for their last visit; 3) the percentage of OOPE by types of services (i.e. healthcare provider fees, medicines, medical tests, transport or other); 4) the proportion of households that incurred catastrophic health expenditure (CHE). The first three of these indicators were derived from individual questionnaires, while the indicator for CHE was derived from household questionnaires.

We defined a household as incurring CHE when OOPE on health exceeded 20, 30%, or 40% or more of a household’s capacity to pay [26]. Capacity to pay was defined as total household expenditure minus subsistence expenditure. To generate subsistence expenditure, the mean of the per capita food expenditure (based on consumption equivalents) of all households between the 45th and 55th percentiles of food as a share of total household expenditure was used. The number of consumption equivalents was calculated using a consumption equivalence scale [27]. We removed household observations for which their total household expenditure was incomplete or equal to zero (5.3% of the sample size). All monetary data are presented in local currency Chinese Yuan.

#### Access

The access domain included: 1) the percentage of respondents who received healthcare the last time it was needed; 2) the percentage of respondents who indicated that cost was a barrier to receiving healthcare; 3) the proportion of respondents who received any outpatient care in the last year or inpatient care in the past 3 years; 4) the number of inpatient and outpatient facilities visited last year; and 5) the proportion of respondents who spent more than 1 h traveling to an outpatient clinic or hospital.

#### Patient-Centredness

We included 10 indicators in the patient-centredness domain for each of outpatient and inpatient care to assess: 1) promptness of care; 2) being treated respectfully; 3) clarity of communication; 4) involvement in decision making; 5) confidentiality; 6) choice of provider; 7) facility cleanliness; 8) overall satisfaction with care received; 9) health condition improvement following care; and 10) achievement of expected outcome.

Responses for these indicators had five levels: 1 = very good, 2 = good, 3 = moderate, 4 = bad, 5 = very bad, except the response for 10) “achievement of expected outcome”, which was a binary indicator (yes/no). The ordinal responses were then dichotomized into “1” (if response was “very good” or “good”) and 0 (if response was “moderate” or “bad” or “very bad”).

#### Equity

We used the relative index of inequality (RII) [28] to measure levels of socioeconomic equity (between the affluent and deprived group) in health system performance indicators in above four domains. RII measures the relative inequality, assuming a linear association between the outcome indicators and ranked economic groups [29, 30]. The value of RII farther away from 1 indicates a larger inequality between the most affluent and the most deprived population in the indicator assessed.

### Statistical analysis

We summarized the mean or median for continuous indicators, the proportion for categorical indicators in the urban and rural areas, respectively. We used the Kruskal-Wallis test (for continuous outcomes variable) and the chi-square test (for binary outcome variables) to compare characteristics between rural and urban respondents. All statistical analyses were conducted using STATA/ SE 14.0.

We used multiple logistic and linear regression models for binary and continuous outcomes, respectively, to assess the differences between urban and rural settings across the first four domains. The numeric indicators in the regression models were log-transformed to normalize the distribution. Urban respondents were the reference group for all analysis, and all regression models run for the first four domains were adjusted for socio-demographic variables (i.e. age, gender, education, income quintile, marital status, insurance status and province of residence). To obtain nationally representative estimates, all data analysis using individual or household level data was weighted to account for the complex, multi-stage sampling strategy of the SAGE survey.

For the equity domain, we used RII to measure the equity between the highest and lowest income group within rural and urban areas respectively. We adopted the widely used Modified Poisson Regression model suggested by Zou [31–33] to measure the relative difference for the binary outcomes of interest. To calculate RII, each income quintile was assigned a ridit score (ranging from 0 to 1) as the explanatory variable. The ridit score was the midpoint of ascending cumulative distribution of each income group (lowest to highest income).

## Results

### Sample characteristics

A total of 13,271 respondents from 9650 households were included in the study; 52.3% of the total sample were rural dwellers, and 47.7% were urban dwellers. Additional file 1 shows the sample flowchart for all outcome variables. Compared to urban areas, rural areas had a younger mean age (61.7 vs 63.5 years), lower percentage of female population (47% vs 54%), less education attained (tertiary or higher: 0.1% vs 9.3%; primary school or less: 80% vs 44.9%), and larger low-income groups (the most affluent quintile: 8.6% vs 36%; the most deprived quintile: 23.1% vs 8.8%). Appendix Table 1 presents the detailed sociodemographic characteristics of the sampled population.

### Effectiveness domain

Overall, respondents living in rural areas fared adversely in nearly all indicators in the effectiveness domain compared to respondents in urban areas (Table 1). The only exception was in treatment of cataracts where a similar proportion of patients in rural and urban areas had an operation.

**Table 1:** Effectiveness domain indicators in rural and urban China

Effectiveness indicator	Overall	Urban (Ref)	Rural	AOR* (95% CI)
Undiagnosed hypertension (%)	56.2	41.4	68.3	**2.76 (**2.35, 3.24)
Take prescribed meds with known HT in the past 12 months (%)	37.8	52.9	25.4	**0.33** (0.28, 0.39)
Hypertension uncontrolled (%)	79.8	68.8	88.8	**2.94** (2.24, 3.86)
Take prescribed meds for diabetes in the past 12 months (%)	83.4	85.3	77.6	0.49 (0.21, 1.16)
Special diet/ weight control for diabetes (%)	67.6	74.6	50.6	**0.37** (0.22, 0.62)
Take prescribed meds for depression in the past 12 months (%)	35.3	45.6	20.9	0.73 (0.05,10.47)
Breast screening coverage in the past three years (%) †	19.3	26.8	11.7	**0.44** (0.30, 0.64)
Cervical screening coverage in the past three years (%) ††	27.5	35.9	18.2	**0.49** (0.29, 0.83)
Eye examination in the past three years (%)	20.4	29.0	8.4	**0.26** (0.13, 0.53)
Operation if have cataracts (%)	22.5	22.0	24.0	1.38 (0.83, 2.31)
Medication or treatment from a dentist in the past 12 months (%)	36.8	50.9	28.3	**0.36** (0.23, 0.56)

†: Breast cancer screening covered women aged 50-74 years old;

††: Cervical cancer screening covered women aged 25-69 years old;

Urban is the reference group.

*All models adjusted for age, gender, education, income quintile, marital status, insurance status and province of residence;

Bold for significance level of 0.05;

In the context of hypertension, the odds of having undiagnosed hypertension was higher among respondents from rural areas than those living in urban areas (68.3% vs 41.4%, AOR = 2.76; 95% CI: 2.35, 3.24). The odds of taking prescribed medicines was lower in rural areas compared with their urban counterparts (25.4% vs 52.9%, AOR = 0.33; 95% CI: 0.28, 0.39), and having hypertension uncontrolled was 2.9 times more likely (88.8% vs 68.8%; AOR = 2.94, 95% CI: 2.24, 3.86) than the urban respondents.

For patients with diabetes, more respondents from urban areas reported taking medication for their conditions (85.3%) compared with their rural counterparts (77.6%). In addition, rural residents had a lower adherence (50.6% vs 74.6%, AOR = 0.37; 95% CI: 0.22, 0.62) to a special diet and weight management to control blood sugar levels than urban residents.

The proportion of women who had breast cancer screening in the past 3 years was low in both rural and urban areas (11.7% vs 26.8%), however, the rural groups were less likely to have recommended levels of breast cancer screening compared to their urban counterparts (AOR = 0.44, 95% CI: 0.30, 0.64). The percentage of women who reported having pap smears for cervical cancer screening was also at a low level in rural and urban areas (18.2% vs 35.9%), however, women from rural areas were still less likely to be screened than those from urban areas (AOR = 0.49; 95% CI: 0.29, 0.83).

Rural residents less frequently had eye examinations (8.4% vs 29%; AOR = 0.26, 95% CI: 0.13, 0.53); operation of cataracts was nonetheless comparable between respondents from rural and urban areas. The odds of having dental treatment for mouth or teeth problems was 64% lower (28.3% vs 50.9%; AOR = 0.36, 95% CI: 0.23, 0.56) among rural residents compared to their urban counterparts.

### Cost domain

The proportion of participants who reported that their last outpatient visit was free was lower in the rural than in urban groups (6.3% vs 10.7%; AOR = 0.48, 95% CI: 0.28, 0.81) (Table 2). However, the mean OOPE for the last outpatient visit for those who reported non-free care was substantially higher in the urban than rural groups (295 vs 139 Yuan; regression coefficient for the log-linear model = − 0.57; 95% CI: − 0.85, − 0.28). In the inpatient setting, while the proportion of participants reporting free inpatient care for the last visit was comparable between rural and urban areas (3.5% vs 4.8%; AOR = 0.92, 95% CI: 0.45, 1.90), mean OOPE for the last inpatient visit remained higher in urban than rural areas (6722 Yuan vs 4194 Yuan for rural residents; regression coefficient for the log-linear model = − 0.38; 95% CI: − 0.69, − 0.06).

**Table 2:** Healthcare costs domain indicators in rural and urban China

Cost indicator	Overall	Urban (Ref)	Rural	Regression coefficients*
**Outpatient**				
The last outpatient visit was free (%)	8.2	10.7	6.3	**0.48** (0.28, 0.81) ^a^
Mean out-of-pocket spending (Yuan)	206	295	139	**−0.57** (− 0.85, − 0.28) ^c^
Median of out-of-pocket spending (Yuan)	60	100	36	NA
Type of spending as % of out-of-pocket expenditure				
Provider fees	4.6	9.3	1.7	**−0.08** (0.02) ^b^
Medicines	82.7	69.5	91.1	**0.28** (0.08) ^b^
Test	8.3	13.0	5.2	**−0.10** (0.03) ^b^
Transport	2.9	4.7	1.8	**−0.04** (0.02) ^b^
Other	1.5	3.5	0.2	**−0.05** (0.02) ^b^
**Inpatient**				
The last inpatient visit was free (%)	4.1	4.8	3.5	0.92 (0.45, 1.90) ^a^
Mean out-of-pocket spending (Yuan)	5397	6722	4194	**−0.38** (−0.69, − 0.06) ^c^
Median of out-of-pocket spending (Yuan)	2000	2500	1800	NA
Type of spending as % of out-of-pocket expenditure				
Provider fees	12.8	13.7	12.0	−0.02 (0.02) ^b^
Medicines	55.2	48.6	60.1	**0.13** (0.03) ^b^
Test	18.2	20.4	16.5	−0.03 (0.02) ^b^
Transport	3.2	5.1	1.7	**−0.04** (0.01) ^b^
Other	10.7	12.1	9.7	−0.02 (0.02) ^b^
**Catastrophic health expenditure (%)**^d^				
20%	37.3	37.3	37.4	**1.30** (1.16, 1.44) ^a^
30%	28.6	27.5	29.4	**1.16** (1.04, 1.30) ^a^
40%	21.9	19.9	23.6	1.08 (0.96, 1.23) ^a^

Out-of-pocket spending was log-transformed to normalize its distribution and used in the regression model

Urban is the reference group

NA: not applicable

*All models adjusted for age, gender, education, income quintile, marital status, insurance status and province of residence;

Bold for significance level of 0.05;

^a^: Odds ratio with 95% confidence interval was presented

^b^: coefficient from linear-regression model with standard error was presented

^c:^ coefficient from log-linear regression model with 95% confidence interval was presented

^d^: percentage of respondents from a household which incurred catastrophic health expenditure

We found that the highest proportion of spending in outpatient and inpatient settings was on medicines, followed by spending on test and provider fees. The rural groups spent a considerably higher proportion of their OOPE on medicines for both outpatient (91.1% vs 69.5%) and inpatient visits (60.1% vs 48.6%), compared to the urban groups.

Households incurring CHE is considerably high in both rural and urban areas (rural vs urban with the threshold at 40, 30 and 20%: 21.4, 27.3 and 35.2% vs 20.6, 27.6 and 37.1%). Similarly, the percentage of individual participants from households incurring CHE ranged from 21.9 to 37.3%. Residents from the rural areas were more likely to be in a household which encountered CHE than their urban counterparts when the CHE threshold was set at 20% (AOR = 1.30; 95% CI: 1.16, 1.44) and 30% (AOR = 1.16, 95% CI: 1.04, 1.30) of the household’s capacity to pay.

### Access domain

This domain evaluated access to outpatient and inpatient care in three aspects (Table 3). Despite the majority of respondents reporting receiving care when needed (95.2% in rural areas; 93.7% in urban areas), a substantial proportion of participants reported cost as a barrier to accessing healthcare (20.6% in rural areas and 26.0% in urban areas).

**Table 3:** Healthcare access domain indicators in rural and urban China

Access indicator	Overall	Urban (Ref)	Rural	Regression coefficient*
Received healthcare last time when needed (%)	94.5	93.7	95.2	1.15 (0.75, 1.78) ^a^
Cost was a barrier to get healthcare (%)	23.5	26.0	20.6	0.82 (0.36, 1.87) ^a^
**Outpatient**				
Any outpatient visits in the past 12 months (%)	60.2	56.8	63.3	1.25 (0.95, 1.64) ^a^
Number of outpatient visits in the past 12 months	5.3	6.3	4.6	−0.18 (0.10) ^b^
How long it took you to get to the clinic (% > 1 h)	2.6	2.2	3.0	0.89 (0.36, 2.34) ^a^
**Inpatient**				
Any hospital stays in the past 3 years (%)	22.2	22.5	21.8	0.93 (0.75, 1.16) ^a^
Length of hospital stays in the past 12 months	0.18	0.19	0.18	−0.02 (0.02) ^b^
How long it took you to get to the hospital (% > 1 h)	7.2	1.2	13.6	**9.34** (3.67, 23.77) ^a^

Number of outpatient visits and hospital stays were log-transformed to normalize its distribution and used in the regression model

Urban is the reference group;

*All models adjusted for age, gender, education, income quintile, marital status, insurance status and province of residence;

Bold for significance level of 0.05;

^a^: regression coefficient represents the adjusted odds ratio (AOR) from the logistic model

^b^: regression coefficient represents the results from linear-regression model

The number of outpatient visits was higher in urban than rural areas (6.3 visits vs 4.6 visits in the last year respectively). However, this difference in number of outpatient visits was not statistically significant (*P* > 0.05) after regression analysis adjusting for covariates.

Only a small proportion of patients from both rural and urban areas spent more than 1 h traveling to the clinic (3.0% vs 2.2%). The number of hospital stays were comparably short in both rural and urban residents (0.18 vs 0.19 in the last year respectively), however, a substantially higher (13.6% vs 1.2%, AOR = 9.34, 95% CI: 3.67, 23.77) percentage of respondents from rural area reported spending more than 1 h travelling to a hospital for inpatient care, compared to the urban population.

### Patient-centredness domain

Among all indicators considered, the proportion of respondents who reported better outcomes in patient-centredness (i.e. “very good” or “good” rating) was overall higher in rural than urban areas (average value of all indicators: 84% vs 82% for outpatient setting; 86% vs 77% for inpatient setting) (Table 4).

**Table 4:** Patient-centredness domain indicators in rural and urban China

Patient-centredness indicator	Overall	Urban (Ref)	Rural	AOR* (95% CI)
**Outpatient**				
Prompt attention	81.8	77.1	85.6	**1.69** (1.16, 2.44)
Respect	85.7	83.9	87.0	1.67 (0.93, 1.77)
Clarity of communication	80.4	80.0	80.7	0.98 (0.73, 1.31)
Involvement in decision making	77.7	76.9	78.3	1.16 (0.84, 1.61)
Confidentiality	77.5	77.6	77.4	1.12 (0.82, 1.53)
Choice of provider	84.2	82.7	85.4	1.29 (0.89, 1.86)
Facility cleanliness	79.8	78.7	80.6	1.11 (0.77, 1.61)
Satisfaction	86.1	83.8	87.9	**1.53** (1.05, 2.21)
Condition improved	90.0	89.6	90.4	1.24 (0.63, 1.89)
Outcome expected (%)	90.6	90.1	91.0	1.30 (0.83, 2.04)
**Inpatient**				
Prompt attention	80.6	73.8	87.1	**2.56** (1.53, 4.29)
Respect	82.0	77.1	86.8	**2.15** (1.23, 3.77)
Clarity of communication	78.8	72.5	85.0	**2.28** (1.27, 4.11)
Involvement in decision making	72.8	66.2	79.2	**2.28** (1.39, 3.74)
Confidentiality	75.9	71.7	79.9	**1.78** (1.12, 2.83)
Choice of provider	80.6	76.9	84.2	**1.76** (1.04, 2.99)
Facility cleanliness	79.0	74.6	83.3	**1.97** (1.24, 3.14)
Satisfaction	82.0	76.3	87.2	**1.81** (1.16, 2.84)
Condition improved	92.6	91.7	93.5	1.31 (0.73, 2.35)
Outcome expected (%)	88.0	85.0	90.7	**1.73** (1.03, 2.90)

Urban is the reference group;

*All models adjusted for age, gender, education, income quintile, marital status, insurance status and province of residence;

Bold for significance level of 0.05;

In the outpatient setting, responsiveness was mostly comparable between rural and urban areas, except two indicators where rural areas performed better: prompt attention to get health service (AOR = 1.69, 95% CI: 1.16, 2.44); and satisfaction with the care received (AOR = 1.53, 95% CI: 1.05, 2.21).

In the inpatient setting, respondents in rural areas perceived better patient-centredness for most indicators compared to their urban counterparts, except for one indicator which was comparable. Compared to urban respondents, the odds of giving a positive rating among rural respondents was more than twice higher than urban respondents in four aspects: prompt attention to patient (AOR = 2.56, 95% CI: 1.53, 4.29), respectfulness to patient (AOR = 2.15, 95% CI: 1.23, 3.77), patient involvement in decision making for treatment (AOR = 2.28, 95% CI: 1.39, 3.74) and clearer communication with healthcare providers (AOR = 2.28, 95% CI: 1.27, 4.11). Rural areas also had better performance regarding clean facilities (AOR = 1.97), satisfaction with inpatient care (AOR = 1.81), confidentiality of consultation (AOR = 1.78) and choice of care provider (AOR = 1.76).

A considerable percentage of respondents in rural and urban areas reported their health condition getting better as expected after outpatient and inpatient care. The odds of achieving the expected outcome from inpatient care was 1.73 (95% CI: 1.03, 2.90) times higher among rural than urban residents.

### Equity domain

Pro-rich inequalities were observed in many effectiveness indicators (Table 5). For example, the RII for the rate of undiagnosed hypertension was less than 1 in both rural and urban areas, indicating that this measure was lower for those with greater wealth. The RII was greater than 1 for rates of breast and cervical screening coverage, indicating that these measures were greater among those with greater wealth.

**Table 5:** Equity domain indicators for effectiveness, cost and access in rural and urban China

Equity index	RII^**∁**^ (95% CI)				Absolute difference
	Urban		Rural		
**Effectiveness**					
Undiagnosed hypertension	**0.77**	(0.65, 0.91)	**0.83**	(0.75, 0.92)	0.06
Take prescribed meds with known hypertension	1.15	(0.98, 1.35)	**1.62**	(1.21, 2.18)	0.47
Hypertension uncontrolled	1.01	(0.85, 1.20)	1.04	(0.97, 1.12)	0.03
Take prescribed meds for diabetes	0.99	(0.81, 1.20)	0.99	(0.76, 1.30)	0
Special diet/ weight control for diabetes	1.02	(0.81, 1.28)	0.64	(0.26, 1.58)	−0.38
Take prescribed meds for depression	1.94	(0.40, 9.54)	1.89	(0.17, 21.34)	−0.05
Breast screening coverage	**2.97**	(1.66, 5.29)	**3.47**	(1.74, 6.91)	0.5
Eye examination	**2.26**	(1.29, 3.96)	**2.83**	(1.19, 6.73)	0.57
Cervical screening coverage	1.64^∘^	(0.83, 3.22)	**7.45**	(2.78, 19.95)	5.81
Operation if have cataracts	0.85	(0.50, 1.43)	1.1	(0.35, 3.50)	0.25
Medication or treatment from a dentist	1.06	(0.68, 1.67)	1.23	(0.80, 1.89)	0.17
**Cost**					
The last outpatient visit was free	1.92	(0.82, 4.52)	1.59	(0.85, 2.92)	−0.33
The last inpatient visit was free	4.63	(0.88, 24.36)	**4.89**	(1.54, 14.73)	0.26
Catastrophic health expenditure					
20%	**0.40**	(0.35, 0.46)	**0.60**	(0.51, 0.71)	0.2
30%	**0.32**	(0.26, 0.38)	**0.52**	(0.43, 0.63)	0.2
40%	**0.24**	(0.20, 0.29)	**0.48**	(0.39, 0.60)	0.24
**Access**					
Received healthcare last time when needed	**1.11**	(1.05, 1.17)	1.03	(0.99, 1.07)	−0.08
Cost was a barrier to getting healthcare	**0.14**	(0.06, 0.32)	**0.15**	(0.03, 0.92)	0.01
Any outpatient visits in the past 12 months	**1.47**	(1.18, 1.83)	1.22	(0.98, 1.51)	−0.25
Time to get to the clinic> 1 h	**0.17**	(0.04, 0.75)	**0.26**	(0.09, 0.77)	0.09
Any hospital stays in the past 3 years	1.17	(0.84, 1.63)	1.11	(0.79, 1.56)	−0.06
Time to get to the hospital> 1 h	**0.09**	(0.01, 0.87)	0.60	(0.17, 2.1)	0.51

RII= Relative Index of Inequality

^∁^: All measures adjusted for respondents’ age and gender unless stated

^∘^: means the result did not adjust for gender;

Bold for significance level of 0.05;

The lowest income group was the reference group with rural and urban areas;

Absolute difference was rural deducted by urban

In indicators for the effectiveness domain, inequalities were wider for rural respondents compared to urban respondents in the following indicators: (a) taking prescribed medicines with known hypertension (RII = 1.62, 95% CI: 1.21, 2.18 vs RII = 1.15, 95% CI: 0.98, 1.35); (b) undertaking breast cancer screening (RII = 3.47, 95% CI: 1.74, 6.91 vs RII = 2.97, 95% CI: 1.66, 5.29); (c) having eye examination (RII = 2.83, 95% CI: 1.19, 6.73 vs RII = 2.26, 95% CI: 1.29, 3.96); and (d) conducting cervical cancer screening (RII = 7.45 95% CI: 2.78, 19.95 vs RII = 1.64, 95% CI: 0.83–3.22).

In the cost domain, both rural and urban areas had pro-rich inequalities in the occurrence of CHE (threshold defined from 20 to 40%: RII = 0.48–0.60 in rural; RII = 0.24–0.40 in urban), indicating that the richer groups had lower risk of incurring CHE. The scale of wealth inequalities in the incurrence of CHE was wider among urban respondents than rural respondents.

Inequality in access to healthcare was in general smaller in rural than urban areas**,** including access to healthcare when needed (RII = 1.03, 95% CI: 0.99, 1.07 vs RII = 1.11, 95% CI: 1.05, 1.17), outpatient visits in the past 12 months (RII = 1.22, 95% CI: 0.98, 1.51 vs RII = 1.47, 95% CI:1.18, 1.83) and time to get to clinic > 1 h (RII = 0.26, 95% CI: 0.09, 0.77 vs RII = 0.17, 95% CI:0.04, 0.75) / hospital > 1 h (RII = 0.60, 95% CI: 0.17, 2.1 vs RII = 0.09, 95% CI: 0.01, 0.87). Nevertheless, inequality in ‘cost as a barrier to access to healthcare’ was comparably large in both rural (RII = 0.15) and urban (RII = 0.14) areas.

Perception of patient-centredness was in general similar among the richest and poorest population groups in both rural and urban areas, except for a few indicators showing statistically significant but minor inequalities for the outpatient setting (Table 6): for example, the widest pro-rich inequality was found in satisfaction with outpatient care received in urban areas (RII = 1.26, 95% CI: 1.11, 1.43).

**Table 6:** Equity domain indicators for patient-centredness in rural and urban China

Equity index	RII^**∁**^ (95% CI)				Absolute difference
	Urban		Rural		
**Outpatient**					
Prompt attention	0.97	(0.84, 1.12)	1.00	(0.91, 1.12)	0.03
Respect	1.01	(0.92, 1.11)	1.01	(0.91, 1.12)	0
Clarity of communication	1.05	(0.95, 1.16)	1.05	(0.92, 1.20)	0
Involvement in decision making	**1.19**	(1.02, 1.40)	**1.12**	(1.00, 1.26)	−0.07
Confidentiality	1.16	(0.98, 1.36)	**1.15**	(1.02, 1.31)	−0.01
Choice of provider	1.10	(0.95, 1.28)	1.03	(0.91, 1.16)	−0.07
Facility cleanliness	1.05	(0.89, 1.24)	**1.16**	(1.01, 1.34)	0.11
Satisfaction	**1.26**	(1.11, 1,43)	1.06	(0.99, 1.34)	−0.2
Condition improved	1.02	(0.91, 1.16)	**0.94**	(0.89, 0.99)	−0.08
Outcome expected	**1.19**	(1.11, 1.28)	1.01	(0.95, 1.07)	−0.18
**Inpatient**					
Prompt attention	0.92	(0.72, 1.18)	0.94	(0.82, 1.07)	0.02
Respect	1.02	(0.83, 1.25)	1.04	(0.92, 1.18)	0.02
Clarity of communication	0.95	(0.76, 1.20)	1.09	(0.92, 1.28)	0.14
Involvement in decision making	1.07	(0.86, 1.32)	1.07	(0.92, 1.24)	0
Confidentiality	1.00	(0.81, 1.25)	1.17	(0.98, 1.41)	0.17
Choice of provider	1.05	(0.81, 1.35)	1.03	(0.90, 1.19)	−0.02
Facility cleanliness	1.08	(0.85, 1.37)	1.12	(0.88, 1.42)	0.04
Satisfaction	1.05	(0.84, 1.32)	1.10	(0.95, 1.25)	0.05
Condition improved	1.06	(0.97, 1.16)	1.04	(0.92, 1.17)	−0.02
Outcome expected	1.11	(0.96, 1.28)	1.09	(0.95, 1.24)	−0.02

*RII=* Relative Index of Inequality

^∁^: All measures adjusted for respondents’ age and gender unless stated

Bold for significance level of 0.05;

The lowest income group was the reference group with rural and urban areas;

Absolute difference was rural deducted by urban

## Discussion

This study examined differences in health system performance between rural and urban areas in China using a large, nationally representative dataset of adults aged 50 years and older.

Our findings that the management and control of hypertension and diabetes were worse in rural than urban areas are consistent with recent studies in China. The awareness, treatment and control of hypertension [34, 35] and diabetes [16, 36, 37] were lower among patients from rural areas especially those with low income, compared to those from urban areas. This is likely to be due to differences in modifiable lifestyle factors (e.g. smoking, alcohol consumption, physical inactivity) associated with hypertension, as well as the lower availability of and higher costs to adhere to prescribed medicines for hypertension among rural groups [34, 35, 38]. In addition, as suggested by Liu et al., better self-awareness of diabetes among urban residents may also lead to better access to preventive health services and disease management than among rural residents [39].

Our findings indicated that while the coverage for breast cancer and cervical cancer screening were poor among eligible women in both rural and urban areas, coverage was worse among those from rural areas and for the poorest. Using data from the China Chronic Disease and Risk Factor Surveillance System (CCDRFSS), Wang et al. (2013) consistently found a lower coverage of breast cancer screening (mammography) [40] in rural areas than urban areas in China. Another study by Wang et al. (2015) also found the coverage of cervical cancer screening (Papanicolaou test) lower in rural compared to urban areas [41]. The disparity in screening coverage between rural and urban areas may reflect a range of social and other factors that are detrimental to preventive service use, including a level of disadvantage related to educational and employment opportunities, income, and access to health services.

We also concluded that although rural residents paid less OOPE for outpatient and inpatient services than urban residents, people from rural areas were still more likely to incur CHE. Previous findings on rural-urban differences in financial protection and healthcare access are mixed. Wang et al. (2016) and Long et al. (2013) showed lower OOPE among rural residents than urban residents, [42, 43] which is consistent with our findings. When restricting the study population to those with chronic conditions, however, Jian et al. (2010) and Li et al. (2018) found that rural residents with chronic conditions had higher OOPE compared to their urban counterparts [44, 45]. This could be due to insufficient benefit coverage of the rural health insurance schemes, where rural residents with chronic conditions had to face higher co-payment rates compared to urban counterparts [44]. Our findings on CHE were consistent with two previous studies at both national and provincial level, where the occurrence of CHE was high in both rural and urban areas and even higher among rural and the poorest households [4, 46].

Our findings showing comparable health services use between rural-urban respondents were supported by several recent studies which showed gradual reduction in the inequity of health care utilization since 1990s [4, 18, 44, 47]. This may be due to the rapid improvement in public health insurance benefit coverage in the rural area in China since the inception of the new rural co-operative medical scheme in 2002 [18].

Our findings on patient centredness were similar to a recent study (based on data from two provinces in China) showing a higher patient satisfaction among rural residents compared with urban residents who visited the county-level hospitals in 2011 [48]. However, our results were in contrast with another recently published study that found higher outpatient satisfaction among people who visited public hospitals in urban areas than those seeking care in rural areas [49]. It is suggested that the differences in benefit coverage of various insurance plans contribute to the gaps in patients’ perspective of care [50].

Overall, our results indicate that significant gaps exist between rural and urban areas in several key health system indicators despite provision of almost universal health insurance coverage. Our findings on rural-urban differences in health system performance are generally consistent with the existing literature [4, 47, 51]. Based on a trend analysis, Meng et al. showed that patients in rural areas tend to face greater risks of catastrophic health expenditure and be less likely to be hospitalized than urban areas [4]. In a review of evidence on the impact of social health insurance (SHI) reforms on patients, Liu et al. found that SHI reduced patients’ co-payment rate and incentivized care-seeking at higher level facilities and longer stays in hospital, whilst the impact on out-of-pocket expenditures varied by rural and urban areas [51].

### Strengths and limitations

Our study has five important limitations. First, use of self-reported measures of chronic disease and healthcare utilisation may underestimate the prevalence, particularly for older persons and those from lower socioeconomic and educational backgrounds who may be more likely to under-report [52]. Second, while SAGE is internationally recognised as one of the highest quality sources to examine health outcomes for the adult and elderly population, the SAGE questionnaire does not include information on other important health system performance indicators such as reproductive, maternal, neonatal and child health services. Third, poorer people (say in rural areas) may have low OOPE because they do not seek access to care due to financial barriers or seek care at lower level health facilities that require lower cost. It is important to note that financial protection cannot be discussed in isolation from access to care [53]. Fourth, some important indicators of health system performance were unavailable from the survey data, such as indicators for infectious disease outcomes. Future studies should consider exploring a wider range of indicators to reduce reporting bias and for health system performance assessment, or incorporate sensitivity analysis to assess the accuracy and validity of the estimates when comparing two groups with different socioeconomic characteristics [52]. Lastly, our study is cross-sectional in design and is therefore unable to examine variation in rural-urban differences in health system performance over time. Future studies using pooled cross-sectional or longitudinal study designs are warranted to monitor changes in rural-urban comparisons.

### Policy implications

China’s health system has made enormous progress towards UHC since the 2000s, especially in rural areas and western and central regions [51, 54]. This includes major advances in reducing rural-urban inequalities in insurance coverage, maternal and infant mortality rates, and use of outpatient and inpatient care [4, 55]. However, there are considerable concerns about the lack of emphasis given to health promotion and prevention of chronic diseases, quality of healthcare services, efficiency of service delivery and cost escalation [4, 56, 57].

This study reveals systematic differences in health system performance between urban and rural areas in China, with implications for equitable access to health care. This is not surprising [47]. Strategies to move towards UHC, especially in middle income countries that have experienced rapid economic growth alongside growing wealth inequalities, and rapid epidemiological and demographic transitions, like China, must manage a complex mix of patient demands and expectations, political acceptability of health system development trajectories, and affordability of UHC. UHC strategies that have been much lauded, such as that of Thailand, have also structurally embedded inequalities [58]. The critical issue is the development of strategy to reduce these inequalities over time.

One of the recent health reforms to tackle inequalities to fulfil the government’s commitment of UHC by 2020 includes a consolidation of the rural (NCMS) and urban (URBMI) social health insurance schemes [59]. SHI consolidation has been piloted in selected Chinese provinces such as Tianjin, Shandong, Zhejiang and Guangdong in eastern, Qinghai, Chongqing, Ningxia in western China [15]. While national and provincial guidelines are needed based on international and domestic experience, it is essential to have political commitments that reinforce political legitimacy, institutional innovations that overcome structural deficiencies, and a feasible implementation plan that coordinates various local conditions, financing capacity and benefit packages for a successful consolidation of SHI [15, 60].

China will only be able to achieve these if it can address the efficiency and cost escalation concerns in its health system. For example, it will only be politically acceptable to more influential urban populations to more fully cross-subsidise rural populations’ access to health care when the growing availability of resources to the whole health system, together with reduction of wasteful use of resources, enables that to be achieved without an appreciable reduction in access to care in urban areas. Some strategies have been developed to address these concerns such as the “zero-mark-up” policy for essential drugs to control cost escalation, and billions of government investment in public health services especially in rural areas to improve efficiency of resource allocation [61]. Further extended health reforms that are embodied in Healthy China 2030, a government blueprint, are needed to meet the ongoing health problems such as chronic diseases, an aging population, and rising expectations of health [55, 62, 63]. The capacity of primary healthcare system needs further strengthened to manage the rising burden of chronic diseases and health expenditures [62].

Unequal development between rural and urban areas in China is not an issue confined to the health sector, and strategies to reduce health system inequalities will need to be accompanied by reductions in social and economic inequalities. Unless services are universally free, uptake of interventions such as breast cancer screening will inevitably be affected by ability to pay and wealth inequalities between rural and urban areas will inevitably translate to rural-urban inequalities in the uptake of screening. Social inequalities are also marked, and health professional staff perceive a considerable gap between the social status of those working in rural primary care and those working in urban tertiary care [64, 65]. It is unlikely that this perception can be much affected without real changes in the social status of rural residents which is currently institutionalised in the hukou system. Hence the health sector will not be the sole source of policies needed to reduce the inequalities described in this data.

## Conclusion

Using a nationally-representative dataset, our study was one of the first to systematically examine rural-urban differences across a list of key health system performance indicators. Our findings that urban areas have achieved better prevention and management of non-communicable disease than rural areas, but access to healthcare was equivalent. Although rural residents incurred less out-of-pocket expenditures for outpatient and inpatient services than urban residents, they were more likely to have catastrophic expenditures on health. Pro-rich inequalities were larger among rural areas than urban areas in terms of hypertension management and coverage of cancer screening. A better understanding of the causes of the observed variations is needed to develop appropriate policy interventions that address these disparities.

### Supplementary information

**Additional file 1: Table S2.** Indicators for health system performance in rural and urban China. **Figure S1.** Sample flowchart for all outcome variables

## Data Availability

The datasets supporting the conclusions of this article are publicly available in the SAGE website [https://www.who.int/healthinfo/sage/en/].

## Appendix

**Table 1:** Characteristics of the sociodemographic variables of the sampled population

Characteristics	All (*n* = 13,271)	Urban (*n* = 6529)	Rural (*n* = 6742)	*P* value^∫^
Age, mean (SD)	62.5 (9.0)	63.5 (9.3)	61.7 (8.6)	< 0.01
Age group in years, n (%)				< 0.01
50–59	5766 (45.1)	2593 (40.5)	3137 (49.3)	
60–69	3941 (31.8)	1894 (31.5)	2047 (31.9)	
70–79	2784 (18.6)	1628 (23.1)	1156 (14.4)	
80 and above	780 (4.6)	414 (4.9)	366 (4.4)	
Gender, (%)				< 0.01
Male	6241 (49.9)	2913 (46.4)	3328 (53.0)	
Female	7030 (50.1)	3616 (53.6)	3414 (47.0)	
Marital status, n (%)				0.38
Married/cohabiting	11,022 (85.1)	5408 (84.5)	5614 (85.7)	
Single/separated/widowed	2249 (14.9)	1121 (15.5)	1128 (14.3)	
Education, n (%)				< 0.01
Primary school or less	8402 (63.3)	2753 (44.9)	5649 (80.0)	
Secondary school	2595 (19.7)	1760 (45.7)	835 (19.9)	
Tertiary or higher	2274 (17.0)	2016 (9.3)	258 (0.1)	
Income quantile, n (%)				< 0.01
Q1 (lowest)	2649 (16.3)	826 (8.8)	1823 (23.1)	
Q2	2641 (18.2)	886 (11.1)	1755 (24.7)	
Q3	2684 (20.6)	1371 (19.1)	1313 (21.9)	
Q4	2719 (23.3)	1540 (25.1)	1179 (21.7)	
Q5 (highest)	2578 (21.7)	1906 (36.0)	672 (8.6)	
Health insurance coverage, n (%)	11,614 (89.6)	5164 (82.0)	6450 (96.6)	< 0.01
Province, n (%)				< 0.01
Guangdong	1566 (16.1)	780 (24.5)	786 (8.4)	
Hubei	1568 (14.3)	797 (9.3)	771 (18.9)	
Jilin	1686 (6.5)	830 (6.3)	856 (6.7)	
Shanxi	1762 (9.0)	864 (7.0)	898 (10.9)	
Shandong	1902 (25.2)	953 (21.9)	949 (28.2)	
Shanghai	1774 (5.6)	820 (4.3)	954 (6.8)	
Yunnan	1550 (9.8)	699 (11.9)	851 (8.0)	
Zhejiang	1463 (13.4)	786 (14.9)	677 (12.1)	

^∫:^*P*-value was calculated using Kruskal-Wallis test for continuous variables and chi-square test for categorical variables
