# Supplementary material for: Rural and urban differences in health system performance among older Chinese adults: cross-sectional analysis of a national sample
Source: BMC Health Serv Res. 2020 May 4;20:372. doi: 10.1186/s12913-020-05194-6 (PMC7197140; doi:10.1186/s12913-020-05194-6)
Supplement: Supplementary file 1 — Additional file 1: Table S2. Indicators for health system performance in rural and urban China. Figure S1. Sample flowchart for all outcome variables [file 12913_2020_5194_MOESM1_ESM.docx]

**Appendix**

Table 2. Indicators for health system performance in rural and urban China

| **Performance measure** | **Definition of indicator** |
| --- | --- |
| **Effectiveness** | |
| Undiagnosed hypertension | Percentage of respondents having mean SBP**^†^** ≥140 mm Hg or mean DBP**^††^** ≥ 90 mm Hg but not self-reported as diagnosed hypertension |
| Take medicines for hypertension | Percentage of respondents with diagnosed hypertension taking prescribed medicine or treatment for hypertension in the past 12 months |
| Controlled hypertension | Respondents self-reporting diagnosed hypertension but mean SBP≤140 mm Hg and DBP≤ 90 mm Hg |
| Take medicines for diabetes | Percentage of respondents with diagnosed diabetes (excluding diabetes associated with pregnancy) taking insulin or other blood sugar-lowering medications in the past 12 months |
| Special diet/ weight control for diabetes | Percentage of respondents with diagnosed diabetes following a special diet, exercise regime or weight control program for diabetes during the last 2 weeks |
| Take prescribed medicines for depression | Percentage of respondents with diagnosed depression taking any medications or other treatment for it in the past 12 months |
| Breast screening coverage | Percentage of women aged 50-74 years old receiving mammography in the past three years |
| Cervical cancer screening coverage | Percentage of women aged 25-69 years old receiving pap smear examination in the past three years |
| Eye examination | Percentage of respondents receiving eyes examination from a medical professional in the past three years |
| Eye surgery to remove cataract (s) | Percentage of respondents with diagnosed cataracts in the last 5 years doing eye surgery to remove this cataract(s) |
| Medication or treatment for mouth and/or teeth problem | Percentage of respondents with problems with mouth and/or teeth receiving any medications or treatment from a dentist or other oral health specialist in the past 12 months |
| **Healthcare costs** | |
| Catastrophic health expenditure (CHE) | Proportion of out-of-pocket spending on health more than 20%, 30% or 40% of the household’s capacity to pay |
| Free care | Percentage of respondents who reported their last outpatient or inpatient care was free |
| Out-of-pocket spending | The mean or median out-of-pocket spending for all respondents |
| Health care provider fees | Proportion of out-of-pocket spending from health care provider fees for last visit |
| Fees for Medicines | Proportion of out-of-pocket spending from medicines for last visit |
| Fees for test | Proportion of out-of-pocket spending from medical tests for last visit |
| Fees for transport | Proportion of out-of-pocket spending from transport for last visit |
| Fees for others | Proportion of out-of-pocket spending from others for last visit |
| **Healthcare access** | |
| Received healthcare when needed | Percentage of respondents who got the healthcare last time when it was needed |
| Cost was a barrier to getting healthcare | Percentage of respondents who did not get the health care because cost was a barrier |
| Any outpatient/ inpatient visits | Percentage of respondents receiving outpatient or inpatient care in the past 12 months |
| Number of outpatient/ inpatient visits | Number of times respondents visited outpatient or inpatient facilities in the last 12 months |
| Time to clinic/ hospital | Percentage of respondents spending more than 1 hour for last visit to an outpatient clinic or hospital |
| **Patient-centredness^†††^** | |
| Promptness of care | The mean scale of amount of time respondents waiting before being attended to for last time visit |
| Being treated respectfully | The mean scale of respectfulness respondents being treated for last time visit |
| Clarity of communication | The mean scale of clarity of communication between health care providers and respondents for last time visit |
| Involvement in decision making | The mean scale of respondent’s involvement in decisions making for their treatment for last time visit |
| Confidentiality | The mean scale of confidentiality respondent had when talking to healthcare providers for last visit |
| Choice of provider | The mean scale of ease respondent had to see the healthcare provider they are happy with for last visit |
| Facility cleanliness | The mean scale of cleanliness of the health facility |
| Satisfaction | The mean scale of respondent’s overall satisfaction with the care received for last visit |
| Health condition improvement | The mean scale of health outcome change after outpatient or inpatient visits |
| Outcome expected**^††††^** | Percentage of respondents got the outcome or results they expected |

**^†^**: systolic blood pressure

**^††^**: diastolic blood pressure

**^†††^**: range 0 (lowest response)-100 (highest response) (except outcome expectation)

**^††††^**: binary response (yes/no)

Figure 1. Sample flowchart for all outcome variables

Baseline sample size n= 15050

Exclude due to:

- Missing value in covariates:

Age (n= 41);

Marital status (n=10);

Income quintile (n=61);

Insurance (n=25);

- Age younger than 50 years old (n=1642)

Baseline sample each after using exclusion criteria n=13271

Effectiveness:

- Undiagnosed hypertension (n= 7714)
- Take medicines for hypertension (n= 7722)
- Controlled hypertension (n=7783)
- Take medicines for diabetes (n=834)
- Special diet/ weight control for diabetes (n=944)
- Take prescribed medicines for depression (n=78)
- Breast screening coverage (n=5735)
- Cervical cancer screening coverage (n=5778)
- Eye examination (n=4828)
- Eye surgery to remove cataract (s) n=1236)
- Medication or treatment for mouth and/or teeth problem (n=1714)

Cost:

- Free care, Out-of-pocket spending (outpatient n=5848; inpatient n=1475)
- Health care provider fees, Fees for Medicines, Fees for test, Fees for transport, Fees for others (outpatient n=4705; inpatient n=939)
- CHE (n=12601)^∫^

Patient-centredness:

- Promptness of care (outpatient n=5940; inpatient n=1391)
- Being treated respectfully (outpatient n=5933; inpatient n=1391)
- Clarity of communication (outpatient n=5932; inpatient n=1390)
- Involvement in decision making (outpatient n=5935; inpatient n=)1391
- Confidentiality (outpatient n=5930; inpatient n=1391)
- Choice of provider (outpatient n=5934; inpatient n=1391)
- Facility cleanliness (outpatient n=5924; inpatient n=1390)
- Satisfaction (outpatient n=6005; inpatient n=1499)
- Health condition improvement (outpatient n=6004; inpatient n=1497)
- Outcome expected (outpatient n=5992; inpatient n=1503)

Access:

- Received healthcare when needed (n=10,077)
- Cost was a barrier to getting healthcare (n=666)
- Any outpatient/ inpatient visits care (outpatient n=10,204; inpatient n=10,088)
- Number of outpatient/ inpatient visits care (outpatient n=6023; inpatient n=9974)
- Time to clinic/ hospital care (outpatient n=5377; inpatient n=1248)
